# Genotypes and phenotypes of resistance in Ecuadorian *Plasmodium falciparum*

**DOI:** 10.1186/s12936-019-3044-z

**Published:** 2019-12-10

**Authors:** Gabriela Valenzuela, L. Enrique Castro, Julio Valencia-Zamora, Claudia A. Vera-Arias, Petra Rohrbach, Fabián E. Sáenz

**Affiliations:** 1 Centro de Investigación para la Salud en América Latina, Facultad de Ciencias Exactas y Naturales, Pontificia Universidad Católica del Ecuador, Av. 12 de Octubre 1076, Apartado, 17-01-2184 Quito, Ecuador; 2 Ministerio de Salud Pública, Guayaquil, Ecuador; 3 Institute of Parasitology, McGill University, Montreal, Canada

**Keywords:** *Plasmodium falciparum*, Malaria, Ecuador, Resistance, Genotypes, Phenotypes

## Abstract

**Background:**

Malaria continues to be endemic in the coast and Amazon regions of Ecuador. Clarifying current *Plasmodium falciparum* resistance in the country will support malaria elimination efforts. In this study, Ecuadorian *P. falciparum* parasites were analysed to determine their drug resistance genotypes and phenotypes.

**Methods:**

Molecular analyses were performed to search for mutations in known resistance markers (*Pfcrt*, *Pfdhfr*, *Pfdhps*, *Pfmdr1, k13*). *Pfmdr1* copy number was determined by qPCR. PFMDR1 transporter activity was characterized in live parasites using live cell imaging in combination with the Fluo-4 transport assay. Chloroquine, quinine, lumefantrine, mefloquine, dihydroartemisinin, and artemether sensitivities were measured by in vitro assays.

**Results:**

The majority of samples from this study presented the CVMN**T** genotype for *Pfcrt* (72–26), NED**F** S**D**FD mutations in *Pfmdr1* and wild type genotypes for *Pfdhfr*, *Pfdhps* and *k13*. The Ecuadorian *P. falciparum* strain ESM-2013 showed in vitro resistance to chloroquine, but sensitivity to quinine, lumefantrine, mefloquine, dihydroartemisinin and artemether. In addition, transport of the fluorochrome Fluo-4 from the cytosol into the digestive vacuole (DV) of the ESM-2013 strain was minimally detected in the DV. All analysed samples revealed one copy of *Pfmdr1*.

**Conclusion:**

This study indicates that Ecuadorian parasites presented the genotype and phenotype for chloroquine resistance and were found to be sensitive to SP, artemether-lumefantrine, quinine, mefloquine, and dihydroartemisinin. The results suggest that the current malaria treatment employed in the country remains effective. This study clarifies the status of anti-malarial resistance in Ecuador and informs the *P. falciparum* elimination campaigns in the country.

## Background

According to World Health Organization (WHO), 132 million people were at risk of malaria infection in 2015 in the Americas. Between 2010 and 2015 there was an estimated 31% decrease in malaria incidence in this region, as well as a 37% decrease in malaria-related mortality. Nevertheless, approximately 450,000 cases were reported in 2015 in this region [1], 30% from Venezuela, 11% from Colombia and 15% from Peru [1]. Thirty per cent of all cases were caused by *Plasmodium falciparum.* Even though, Ecuador accounted for less than 1% of the region malaria cases in 2015, malaria continues to be endemic in the coast and Amazon areas of Ecuador. From 2016 to 2018, Ecuador has seen increases in malaria cases (1191 in 2016, 1380 in 2017 and 1806 in 2018), where *P. falciparum* was responsible for 10% of these cases [2].

The current treatment for *P. falciparum* infection in the Americas is based on artemisinin in combination with another anti-malarial (ACT) [3]. Treatment for uncomplicated falciparum malaria in Ecuador relies on the combination artemether-lumefantrine + primaquine; for *Plasmodium vivax* the treatment used is chloroquine + primaquine [4]. *Plasmodium falciparum* has developed resistance to almost all available anti-malarials, necessitating the need for an adequate knowledge of anti-malarial drug effectiveness. This is especially true in low transmission areas, where malaria elimination is ongoing, as the inflow of resistant parasites can generate unwanted outbreaks.

Chloroquine (CQ) resistance in *P. falciparum* was reported in 1957 on the Thailand-Cambodian border in Southeast Asia and almost at the same time in Colombia and Venezuela in South America, before spreading to the rest of the world [5]. Mutations in the *P. falciparum* chloroquine resistance transporter (PfCRT) are considered the main reason for CQ resistance [6]. Currently, CQ resistance is found throughout South America [5] and the PfCRT molecular marker K76**T**, thought to be mainly responsible for CQ resistance, is considered fixed in this region [7]. The PfCRT haplotype CVMN**T** (positions 72–76) has been reported in Colombia and Peru, while CVM**ET** and CV**EIT** have been reported in Colombia and Venezuela and **S**VMN**T** has been reported in the Amazon region of Brazil and Peru [8].

During the 1970s the combination sulfadoxine-pyrimethamine (SP) was introduced in South America as treatment against *P. falciparum*. Shortly after the introduction, resistance to these drugs was reported [8]. Colombian, Brazilian and Peruvian parasite isolates showed mutations in *Pfdhps* mainly in positions 437, 540 and 581. The mutation A437**G** is dominant in Colombia, while the mutations A437**G** and K540**E** are found in Peru [7–10]. In addition, Venezuela and Bolivia have reported the mutation K540**E** in 90% of the parasite samples tested [7, 8]. *Pfdhfr* mutations C50**R**, I165**L** and S108**N/T** are common throughout South America [8, 9] and all mutations are associated with SP resistance [11].

*Plasmodium falciparum* multidrug resistance 1 (*Pfmdr1*) transporter gene encodes for a p-glycoprotein that is part of the adenosine triphosphate-binding cassette transporter family. Mutations in *Pfmdr1* are associated with multidrug resistance, and show reduced susceptibility to mefloquine (MQ), halofantrine (HF), quinine (QN), and possibly lumefantrine (LUMF) [11, 12]. The PfMDR1 mutations N86Y and Y184F are common in Asia and Africa, while the mutations S1034C, N1042D and D1246Y are mostly found in South America [13].

Several studies associate increases in *Pfmdr1* copy number to MQ resistance, and QN and CQ susceptibility [13–16]. Recent research suggested that an increase in *Pfmdr1* copy number is related to artemisinin resistance [13, 14]. In South America, there are reports of changes in *Pfmdr1* copy number, specifically in samples coming from the Pacific region, Atlantic region and southeastern Colombia, where an increase of 2 to 5 copies of *Pfmdr1* were found in 30% of the parasite samples [15]. Peru reported single *Pfmdr1* copy numbers [17].

Resistance to artemisinin (ART) in *P. falciparum* has been reported in five Asian countries: China, Vietnam, Cambodia, Thailand, and Myanmar. The current management to control *P. falciparum* infections is based on ART derivatives combined with a partner anti-malarial (e.g., MQ, LUMF, primaquine) [18]. *Kelch 13* (*k13*) propeller mutations have been associated with ART resistance and can be used as molecular markers to monitor the possible emergence of ART resistance [3, 18]. ACT treatment continues to be effective in South America. New studies in Brazilian, Peruvian and Colombian isolates show no *k13* mutations associated with ART resistance [5, 19, 20].

In addition to genetic variability studies, drug resistant phenotypes can be characterized using in vitro assays. In particular, Colombia reported low in vitro susceptibility to CQ and amodiaquine (AQ) in almost 90% of the isolates analysed, showing IC_50_ values for both anti-malarials greater than 100 nM [14, 21]. Furthermore, all samples showed high susceptibility to dihydroartemisinin (DHA), LUMF and artemether (ATM) [14, 21, 22]. Brazilian samples from the Amazon region also showed resistance to CQ and AQ, with an elevated IC_50_ [23]. In vitro assays with field parasites in South America have been limited, since culture adaptation of field parasites to laboratory conditions require a long time and are usually challenging [24].

In 2002, studies from Ecuador reported mutations in isolates collected in Esmeraldas. The parasites presented the CVMN**T** *Pfcrt* genotype and one, (position 108**N**) two (positions 108**N**, 164**L**) or three (51**I**, 108**N**, 164**L**) mutations in *Pfdhfr* [25]. In 2013, parasite isolates showed wild type genotypes for *Pfdhfr* and *Pfdhps*, the CVMN**T** and CVM**ET** *Pfcrt* genotypes, and the mutations Y184**F** and N1042**D** in *Pfmdr1* in an outbreak that occurred in Esmeraldas [26]. These genotypes indicated that Ecuadorian strains were CQ resistant and mostly sensitive to sulfadoxine and pyrimethamine [26]. This genotype was shared with Ecu 1110, a 1990 isolate from the same area. Ecu1110 has an in vitro CQ resistance phenotype (IC_50_ > 90 nM) [27].

In this study, in vitro assays were used to determine drug susceptibility phenotypes. In addition, drug resistance genotypes were analysed in *Pfcrt, Pfdhfr, Pfdhps, pfmdr1,* and *k13* of Ecuadorian *P. falciparum* isolates. The aim of this study was to understand current anti-malarial resistance in Ecuador, in order to support malaria elimination efforts in the country.

## Methods

### Ethics statement

The samples used in this study were obtained by the malaria control and elimination programme and approved by the Ethical Review Committee of *Pontificia Universidad Católica del Ecuador* (CBE-016-2013 and CEISH-163-2016). Written informed consent was provided by study participants and/or their legal guardians.

### Study site and sample collection

**Fig. 1:**
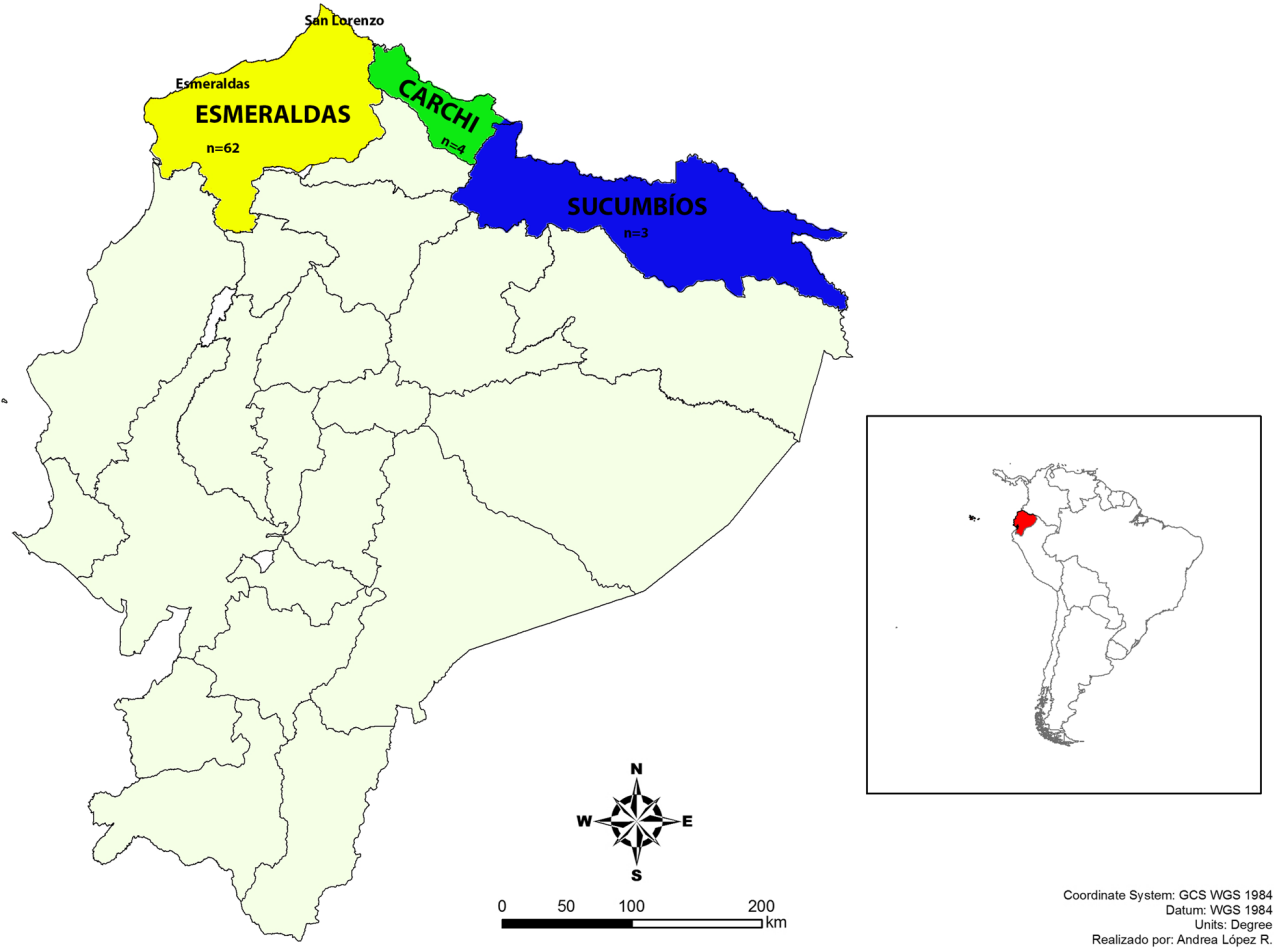
Study site. All samples were collected in three northern provinces of Ecuador: Esmeraldas n = 62 samples, Carchi n = 4 samples and Sucumbíos n = 3 samples

Sixty-nine samples were analysed in this study, 62 of these (89.9%) were collected in Esmeraldas province. Esmeraldas is located in northwest Ecuador and borders with Colombia. All the samples collected in this province came from Esmeraldas and San Lorenzo counties, where the incidence of *P. falciparum* is the highest in Ecuador. Four samples were collected in the Carchi province, located in the north of Ecuador, east of the Esmeraldas province. Three samples were collected in Sucumbíos, a province located in the north Amazon of Ecuador, east of Carchi province (Fig. 1). All samples were collected between 2013 and 2015 through the National Service for Control of Diseases Transmitted by Arthropod Vectors (SNEM). Eighty-five per cent of the samples were collected as whole blood and 15% on filter paper and kept at 4 °C.

### DNA extraction and infection confirmation

The DNA from 69 samples was isolated from filter paper using QIamp DNA mini-spin kit (QIAGEN, Valencia, CA, USA), and from whole blood using Axyprep body fluid viral DNA/RNA Miniprep (AXYGEN, Union City, CA, USA). Confirmation of *P. falciparum* infection was performed by microscopy (slides were stained with Giemsa stain 20%, for 20 min) [28] and nested-PCR using the 18S ribosomal RNA gene. Genomic DNA was used for *Plasmodium* spp. detection. DNA was amplified using nested PCR as previously described [29]. The amplification was done using primers that target the 18S ribosomal of *Plasmodium* spp. The 25 µl reaction contained 2.5 µl of Mg2Cl, 3 µl of water, 12.5 µl of Green GoTaq Master Mix, 1 µl of each primer and 5 µl of DNA [29].

### Analysis of drug resistance markers

*Plasmodium falciparum* 3D7, W2, D6, CAM6 and C2B isolates were used as controls and Ecuadorian *P. falciparum* were analysed to identify drug resistance-associated mutations in *Pfcrt* (positions 72–76), *Pfmdr1* (positions 134, 184, 1034, 1042, 1226, 1246), *Pfdhfr* (positions 51, 59, 108, 164), *Pfdhps* (positions 436, 437, 540, 581, 613), and *k13* (positions 476, 493, 539, 543, 580) using conditions and primers reported previously in other studies [9, 27–29] (Additional file 1: Table S1). All PCR amplicons were visualized on 2% agarose gels and purified with 5 μl of Illustra ExoProStar (GE Healthcare, Piscataway NJ, USA) at 37 °C for 25 min and 20 min at 80 °C. After visual confirmation of amplified product, all amplicons were submitted to MACROGEN, South Korea, for capillary sequencing. Sanger sequencing was performed on all samples to identify drug resistance-associated mutations in *pfcrt, pfdhfr, pfdhps*, *pfmdr1* and *k13* genes.

The sequencing results were analysed and aligned using Geneious software version 10 (Biomatters, Inc, Newark, NJ, USA). The sequences were compared to controls (3D7, W2, D6, CAM6, C2B) to establish which samples present mutations. The sequence alignments were analysed, and SNP differences were established to determine the mutation frequencies for each gene. All mutations frequencies were analysed using Microsoft Excel 16.9.

### *Pfmdr1* copy number

*Pfmdr1* copy number was established with real time PCR, using TaqMan probes for *Pfmdr1* (target gene), and the housekeeping gene *Seryl*-*t*-*rna*-*synthetase* was used as a control.

The primers used for quantifying copy number were: *PF_F:* 5TTAAGTTTTACTCTAAAAGAAGGGAAAACATA,PF*_R:* 5′TCTCCTTCGGTTGGATCATAAAG, seryl_F:5′GATTTATTAAGAAAAATAGGTGGAGCTA, seryl_R:5′TATAGCATTATGTAATAAGAAACCTGC, and the Taqman probes were: *PF_FAM:* 5′FAMCATTTGTGGGAGAATCAGGTTGTGGGAAAT_TAMRA, seryl_probe:5′VICAAGGTATACAAGTAGCAGGTCATCGTGGTT_TAMRA [30].

Each 20 µl of reaction mix contained 15 ng DNA, 300 nM primers, 250 nM TaqMan probes and 10 µl TaqMan Fast Advance Mix from Applied Biosystems (Austin, TX, USA). Triplicates of each sample were analysed using the following amplification protocol initial activation: 94 °C for 3 min, followed by 40 cycles of: 94 °C for 1 min, 60 °C for 1 min, and 72 °C for 30 s. Fluorescence was recorded after each elongation step. Real-time PCR was carried out in StepOne™ Real-Time PCR System (Applied Biosystems). Copy numbers were calculated relative to 3D7, which is known to have only one *Pfmdr1* gene copy; the seryl gene is consistently expressed throughout the parasite blood stages. The copy number of *Pfmdr1* was calculated using relative quantification (RQ) by the calculation of ΔΔCt, using the Ct generated by *Seryl* and *Pfmdr1* for each sample by a comparative threshold method, with the formula ΔΔCt = (Ct _*mdr1*_ − Ct _*seryl*_)_Sample_ − (Ct _*mdr1*_ − Ct _*seryl*_)_3D7_, 2^−(ΔΔCt)^ where Ct is the threshold cycle for each gene [16].

### Phenotype characterization

#### Parasite strains and culture conditions

The standard strains 3D7 (CQ sensitive) and W2 (CQ and SP resistant) were used as controls for in vitro susceptibility assays: *P. falciparum* isolate ESM-2013 was obtained from a patient in Esmeraldas city in 2013 and subsequently adapted to laboratory conditions. Parasites were cultured in human O+ red blood cells, following previously reported methods [31], with modifications. RPMI 1640, supplemented with 25 mM HEPES, 8,9 mM sodium bicarbonate and human O+ plasma was used as complete medium. All parasites were cultured in red blood cells at 4% haematocrit with complete medium at 37 °C and mixed gas (5% O_2_, 5% CO_2_ and 90% of N_2_ or 3% O_2_, 5% CO_2_ and 92% N_2_ according to laboratory conditions).

#### Drug sensitivity assay

In vitro sensitivity assays were performed with the following drugs: chloroquine disphosphate (CQ), dihydroartemisinin (DHA), quinine sulfate (QN), mefloquine hydrochloride (MQ), lumefantrine (LUMF) and artemether (ATM). The drugs were provided by Dennis Kyle, University of South Florida. Drug sensitivity in vitro assays of the parasites was performed by microscopy to establish the IC_50_s of several anti-malarial drugs. Drug stock solutions were prepared in dimethyl sulfoxide (DMSO) or water, at an initial concentration of 1 mg/ml. The experiments were set up in 96-well plates with 2-fold dilutions of each drug across the plate in a total volume of 150 µl and at a final red blood cell concentration of 1.5% (vol/vol). The experiment was started at an initial parasitaemia of 0.5% (80% rings) synchronous parasite-infected red blood cells (PRBC). The plates were incubated for 72 h at 37 °C in an atmosphere of 5% CO_2_, 5% O_2_, and 90% N_2_. A light microscope was used to look for the presence of schizonts in a thick smear to establish parasite growth.

Parasitaemia was determined by counting 1000 red blood cells (RBCs) in thin smears and the number of infected cells (iRBCs) using the equation (No. iRBCs * 100/No. RBCs). All in vitro assays were performed in duplicate with at least two replicates.

Thin blood smears were fixed in methanol and stained with 20% GIEMSA for 20 min. The data were analysed using Excel 16.9 software to compare the parasitaemia of each well versus drug concentration. Parasite IC_50_ curves were obtained using non-linear regression and IC_50_s were calculated by using the equation of the curve. Linear point interception with the curve was used to establish inhibitory concentrations.

### Dye loading and live cell imaging

The laboratory strain Dd2 and the Ecuadorian *P. falciparum* isolate ESM-2013 were used for this experiment. Synchronized trophozoite stage parasites were loaded with 5 µM Fluo-4 AM (Life Technologies, Burlington, ON, Canada) in Ringer’s solution (122.5 mM NaCl, 5.4 mM KCl, 1.2 mM CaCl_2_, 0.8 mM MgCl_2_, 11 mM d-glucose, 10 mM HEPES, 1 mM NaH_2_PO_4_, pH 7.4) for 50 min at 37 °C. Parasites were washed twice with Ringer’s solution and transferred to a microscope chamber, where they were kept at 37 °C during microscopy. A series of images per parasite was taken using a Zeiss LSM710 confocal microscope (Carl Zeiss, Oberkochen, Germany) equipped with a water-corrected objective (C-apochromat 63×/1.20 W Korr M27) and a 488 nm laser (12.5 mW, 2% intensity). The range of emitted fluorescence was measured from 493 to 622 nm. The acquired images were analysed using the Zen software (Carl Zeiss) and the fluorescence averaged [32].

## Results

### Genotyping of drug resistance markers

Sixty-two out of the 69 collected samples were analysed for mutations in five genes: *Pfcrt* (positions 72–76), *Pfdhps* (positions 436, 437, 540, 581, 613), *Pfdrfr* (positions 50, 51, 59, 108, 164), *Pfmdr1* (positions 86, 130, 144, 184, 1034, 1042, 1226, 1246) and *k13* (positions 476, 493, 539, 543, 580). Thirty-two samples from Esmeraldas county and 23 samples from San Lorenzo county (total of 55 samples in Esmeraldas province) were collected, as well as 4 samples in Carchi province and 3 in Sucumbíos province. F50 is a sample collected in Nuevo Rocafuerte, Aguarico, Orellana province in 2014; this province is located on the east border of Ecuador to Peru. In addition, Ecu 1110 was collected in Esmeraldas in 1990, this province is located in northern border and was included in the analysis.

**Fig. 2:**
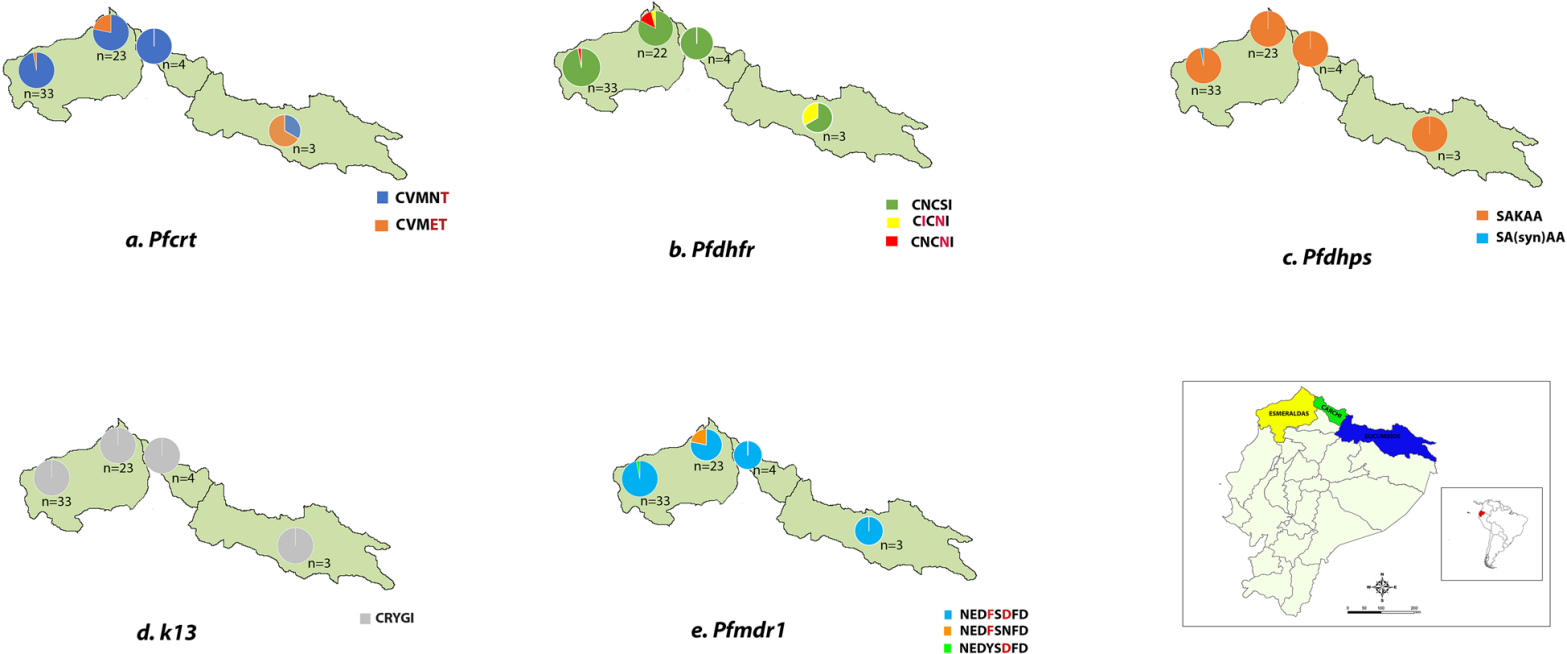
Drug resistance haplotype distribution and frequency in Ecuadorian *Plasmodium falciparum* study area. Distribution of **a** *Pfcrt,* **b** *Pfdhfr,* **c** *Pfdhps*, **d** *k13*, **e** *Pfmdr1* haplotypes

The samples collected in Esmeraldas county presented two mutated haplotypes of *Pfcrt*: 97% of the samples presented the CVMN**T** haplotype with a simple mutation in the position 76 (K76T) and 3% of the samples showed the CVM**ET** haplotype with double mutations at positions 75 (N75**E**) and 76. Thirty-one of these samples were reported in a previous study [26]. In San Lorenzo county, the same haplotypes were found but in different frequencies, 78.3% of the samples presented the haplotype CVMN**T** and 21.3% showed CVM**ET**. All of the samples collected in Carchi showed the CVMN**T** haplotype. This haplotype occurs in 33.3% of Sucumbíos samples, while, 66.7% presented the CVM**ET** haplotype (Fig. 2a). Only the sample F50 collected in Orellana presented the haplotype **S**VMN**T** with double mutations at positions 72 and 76 (C72**S**). The Ecu1110 isolate, collected in Esmeraldas in 1990, also presented the CVMN**T** haplotype for *Pfcrt*. In summary, 100% of the samples analysed showed at least one mutation in the 72–76 positions of *Pfcrt.* All of the genotypes found are associated to CQ resistance (Table 1).

**Table 1:**
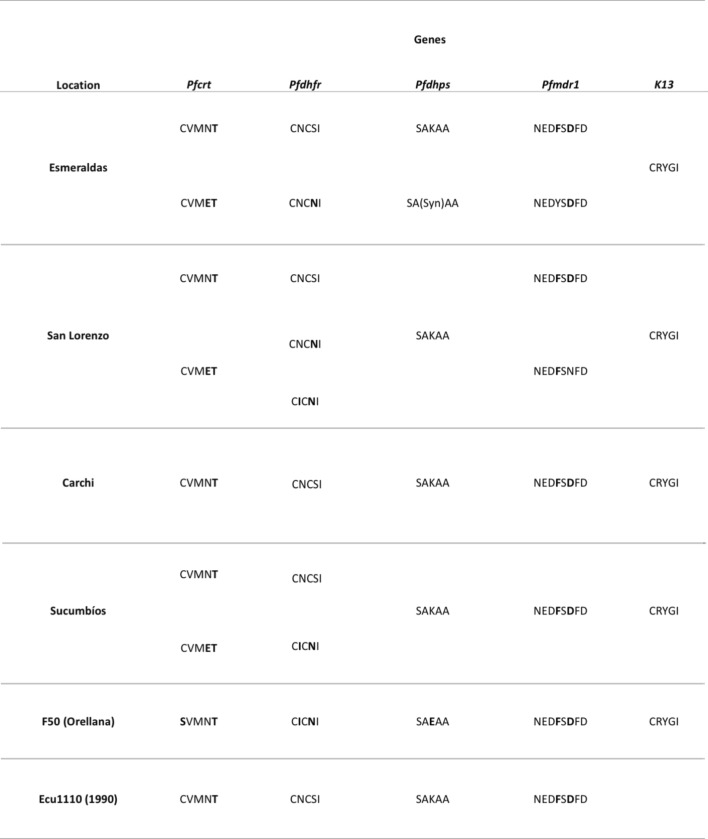
Drug resistance markers haplotypes by location

*Pfdhfr* presented the wild genotype CNCSI in the majority of samples: 97% in Esmeraldas county, 82.6% in San Lorenzo county, 66.7% in Sucumbíos and 100% in Carchi. The CNC**N**I haplotype, with a simple mutation in the position S108**N**, was found in 13.04% of San Lorenzo samples, and the C**I**C**N**I haplotype (double mutant at positions N51**I** and S108**N**) in 4.36% of San Lorenzo samples and 33% of Sucumbíos samples (Fig. 2b). The double mutation (C**I**C**N**I) was also found in F50 (Orellana). In contrast, Ecu 1110 presented the wild type genotype CNCSI. San Lorenzo county showed higher diversity of haplotypes for the *Pfdhfr* gene (Table 1), although, the wild type genotype was the most frequent in the population.

The wild type genotype (SAKAA) of *Pfdhps* was the dominant genotype in all samples analysed: 97% of Esmeraldas samples and 100% of San Lorenzo, Carchi and Sucumbíos samples (Fig. 2c). One sample from Esmeraldas county presented a synonymous mutation in the position 540, the sample F50 from Sucumbíos had the mutation K540**E,** showing the haplotype SA**E**AA (Table 1).

Mutations in the gene *k13* have been related to artemisinin sensitization. This gene was analysed in five separate positions (476, 493, 539, 543, and 580). All the samples studied in Esmeraldas, San Lorenzo, Carchi and Sucumbíos presented the wild type haplotype CRYGI (Fig. 2d).

*Pfmdr1* presented two main mutations: Y184**F** and N1042**D** in the majority of samples analysed in this study. In fact, the NED**F**S**D**FD haplotype was present in 97% of Esmeraldas county samples, 78.3% of San Lorenzo county samples and 100% of Carchi and Sucumbíos samples (Fig. 2e). Surprisingly, the F50 sample and the Ecu 1110 also presented the genotype NED**F**S**D**FD. In contrast, only 3% of Esmeraldas county (F31 sample) presented the mutation 1042 (NEDYS**D**FD) alone, and 21.7% of San Lorenzo county samples presented the mutation in position 184 (NED**F**SNFD). The mutations 184 and 1042 were found in almost all samples (Table 1).

### Copy number of *Pfmdr1*

**Fig. 3:**
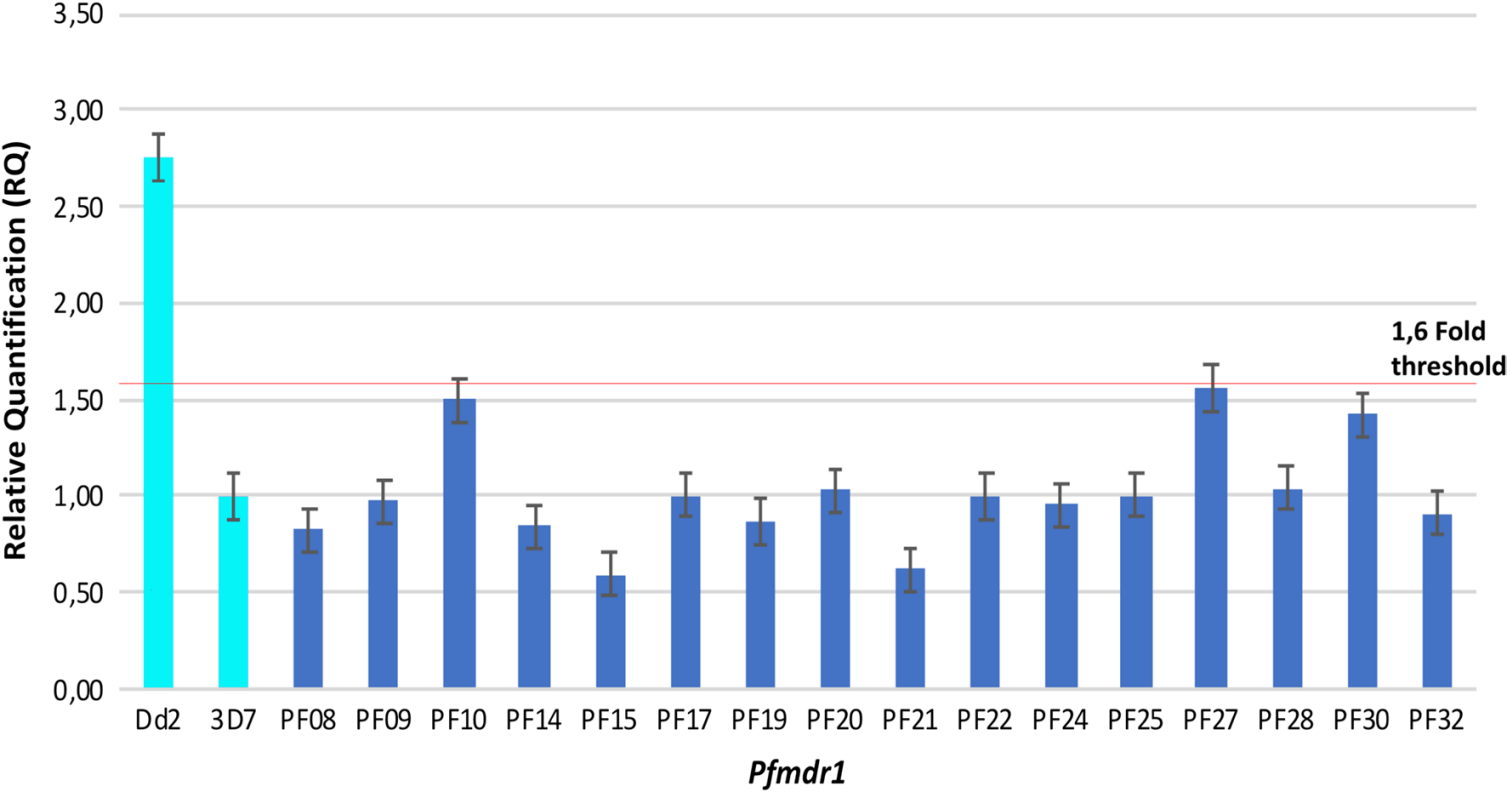
*Pfmdr1* copy number. Dd2 presents two copies and 3D7 one copy. All samples analysed presented one copy of *Pfmdr1*. Sixteen of the 45 analysed samples are shown. Copy number > 1,6 was defined as a duplication of *pfmdr1* [42]

*Pfmdr1* copy number was determined by qPCR. The housekeeping gene *Seryl* was used as an internal control. 3D7 and Dd2 strains were used as copy number controls, where 3D7 showed one copy and Dd2 two copies of the *Pfmdr1* gene [30]. Sixty-two samples were analysed, 75% (45/62) of the samples were amplified by qPCR. One-hundred per cent of the samples collected in Esmeraldas county, San Lorenzo county, Carchi and Sucumbíos presented only one copy of *Pfmdr1* (Fig. 3).

### Live cell imaging

**Fig. 4:**
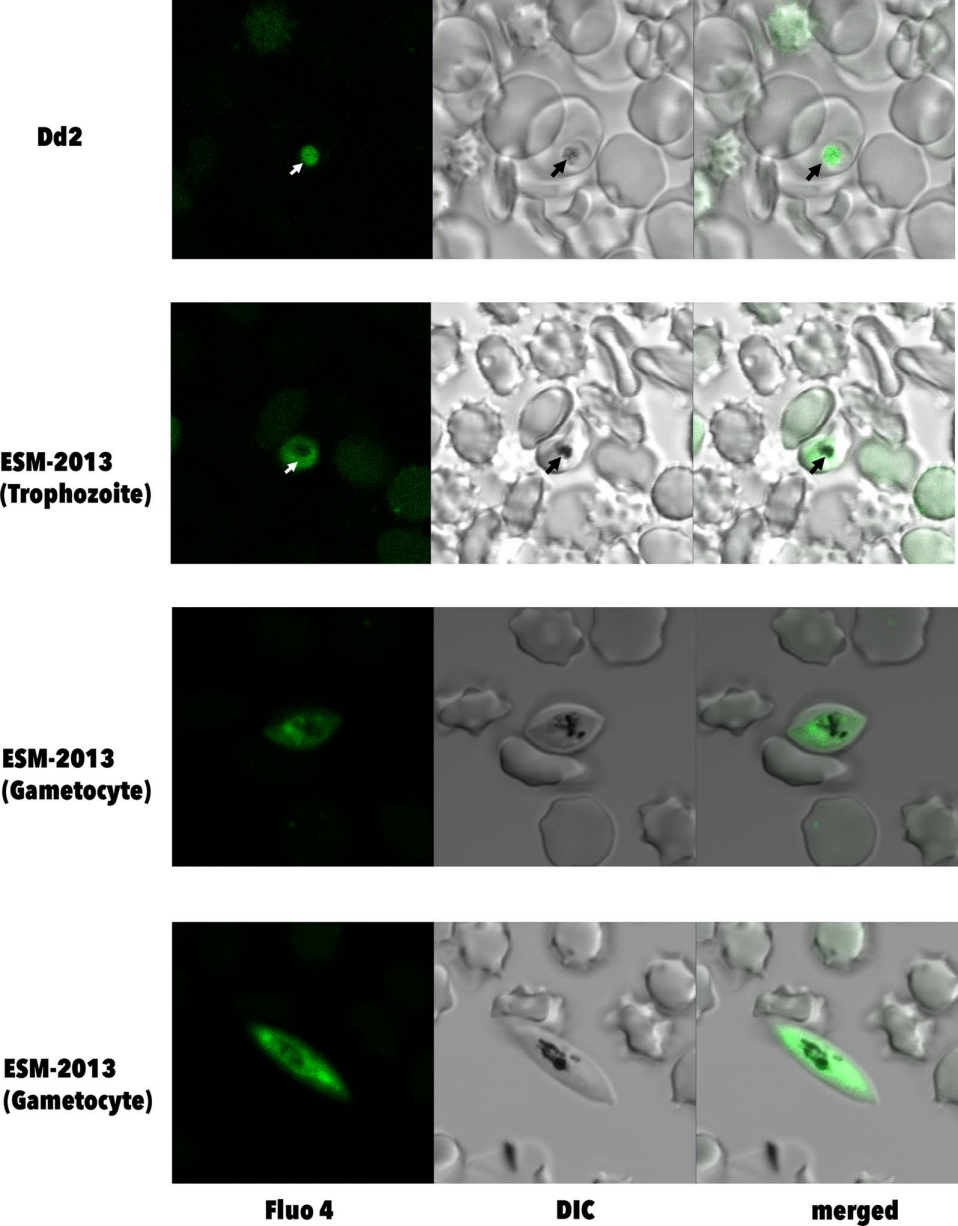
Fluo 4 fluorescence in *Plasmodium falciparum* parasites. Trophozoite stage parasites were incubated with 5 µM Fluo 4 AM. Dd2 parasites accumulate Fluo 4 in the digestive vacuole (DV), while ESM-2013 parasites accumulate Fluo 4 in the cytosol, suggesting a reduced transport of the fluorochrome to the DV. Arrow: digestive vacuole

Ecuadorian *P. falciparum* isolates were analysed for drug resistance haplotypes at eight different amino acid positions as well as copy number variations of *Pfmdr1*. These parasites presented two main mutations: Y184**F** and N1042**D** and only one copy of the gene. Fluo 4 AM is a fluorochrome and is transported by PfMDR1 from the cytosol to the digestive vacuole (DV) [30, 32, 33]. The use of Fluo 4 AM can help investigate the role of the PfMDR1 transporter. The mutation N1042**D** has been correlated to reduced transport of Fluo 4 from the cytosol to the DV of the parasite [32]. The parasite ESM-2013, an isolate from Esmeraldas that presented the mutation N1042**D**, was cultured with Fluo 4 AM and visualized by confocal microscopy to establish the transport of the fluorochrome. Dd2 strain was used as a positive control that allows the transport of Fluo 4 AM to the DV. The results show that Fluo 4 is not transported to the DV (i.e., Fluor-4 fluorescence does not accumulate) in ESM-2013 parasites (Fig. 4). The ratio of Fluo-4 fluorescence intensity (when calculating DV/cytosol Fluo-4 fluorescence measured in these compartments) of Dd2 was 6.9. In contrast, ESM-2013 presented a DV/cytosol Fluo-4 fluorescence ratio of 0.50.

### In vitro drug sensitivity characterization

In vitro assays were used to test the sensitivity of Ecuadorian *P. falciparum* isolates to CQ, MQ, QN, ATM, DHA, and LUMF. ESM-2013 was exposed to anti-malarial drugs to establish its IC_50_. This parasite also presented a genotype associated with CQ resistance (CVMN**T**). The parasite ESM-2013 showed an IC_50_ of 93.71 nM (Table 2). Furthermore, ESM-2013 presented considerably lower IC_50_ for MQ, QN, LUMF, ATM, and DHA. ESM-2013 presented 4.94 nM IC_50_ for MQ, 7.63 nM for QN and 2.60 nM for DHA, indicating a phenotype of sensitivity for those anti-malarial drugs. The current treatment for *P. falciparum* infections in Ecuador is based on ATM and LUMF. This treatment was tested in ESM-2013 and this parasite showed an IC_50_ of 3.59 nM for LUMF and 1.14 nM for ATM (Table 2).

**Table 2:** IC_50_ values of *Plasmodium falciparum* ESM-2013 and 3D7

Drug	ESM-2013	3D7
	IC_50_ [nM]	IC_50_ [nM]
CQ	93.71 ± 33.07	8.36 ± 5.6
MQ	4.94 ± 0.6	19.3 ± 2.15
QN	7.63 ± 1.08	23 ± 1.8
LUMF	3.59 ± 0.4	7.46 ± 2.6
ATM	1.14 ± 0.7	3.1 ± 0.8
DHA	2.6 ± 1.4	2.4 ± 1.3

## Discussion

Ecuador has been very successful in reducing the number of malaria cases in the country. It is estimated that more than 99% prevalence reduction took place from 2000 to 2015 [34]. In this context, understanding drug resistance genotypes and phenotypes of Ecuadorian *Plasmodium* isolates constitutes crucial information for supporting the National Malaria Programme to achieve elimination of the disease in the country.

Ninety per cent of the samples analysed in this study were collected in Esmeraldas province (Esmeraldas and San Lorenzo counties), where most *P. falciparum* cases are known to occur. In 2016, this province reported 125 *P. falciparum* cases, roughly 45% of the total of *P. falciparum* cases in the country [2].

This study determined the mutations associated with five different genes involved in CQ, SP, MQ, QN, and ART resistance present in Ecuador. The results showed that Ecuadorian parasites presented the CQ resistance haplotypes CVMN**T**, CVM**ET** and **S**VMN**T** in *Pfcrt* (72–76). The genotype CVMN**T** was found in Esmeraldas, San Lorenzo, Carchi and Sucumbíos while the CVM**ET** genotype was found in San Lorenzo and Sucumbíos, the **S**VMN**T** genotype was found in one sample from Orellana. CVMN**T** and CVM**ET** genotypes have previously been reported in Colombia [8]. CVMN**T** was reported in the Pacific coast of Peru and the CVM**ET** genotype was reported in the Amazon region of Peru [10] The genotype **S**VMN**T** has been found in the Peruvian [10] and Brazilian Amazon [9].

The CVMN**T** haplotype had been previously reported in a parasite isolate obtained in Esmeraldas in the 1990 [27]. Additionally, this genotype was reported in Ecuador during an outbreak occurred in 2013 [26]. This suggests that this haplotype has been circulating in Ecuador for decades and is still maintained in the country. Griffing and collaborators suggest that parasites carrying this haplotype, were circulating in Colombia and then crossed into Ecuador and later entered Peru [10].

The resistance to CQ is considered fixed in South America, since CVMN**T**, CVM**ET** and **S**VMN**T** are common genotypes in the region [7, 9]. However, in Ecuador, the national treatment regime was changed from CQ in 2004 to artesunate + SP [35] and, more recently, to ATM-LUMF [26]. *Plasmodium falciparum* parasites circulating in the region continue to have the K76**T** genotype of *Pfcrt*. This could be related to continuous drug pressure in the parasite population, since CQ remains the main treatment to control *P. vivax* infections.

The wild type genotype CNCSI for *Pfdhfr* was the main genotype found in all locations sampled in this study (Ecuador, Peru, Colombia, and in the Ecu 1110 isolate) [27]. The genotype with a simple mutation in position 108 (CNC**N**I) was only found in San Lorenzo county. These genotypes have previously been reported in Ecuador [25], Colombia [8] and Peru [10]. The C**I**C**N**I resistance genotype was found in San Lorenzo county and Orellana; Peru, Colombia and Brazil have also reported this genotype [8, 9].

The *Pfdhfr* polymorphisms were more diverse in San Lorenzo than in the other Ecuadorian locations. San Lorenzo is located close to the Colombian border suggesting that the migration of parasites from Colombia to Ecuador could be related to the distribution of these mutations. In fact, double and triple mutations in *Pfdhfr* and resistance to SP have frequently been reported in Colombia [8]. In addition, it has recently been reported using neutral microsatellites that *P. falciparum* populations from San Lorenzo are shared with the south of Colombia [36].

The wild type genotype SAKAA for *Pfdhps* presented the highest frequency in all studied locations, as well as for Ecu 1110 [27]. Only the sample F50 from Orellana presented mutation in this position (SA**E**AA). This mutation is common in Brazil [9], Venezuela, Bolivia, and Peru [8, 10]. The wild type genotype was previously found in samples from Esmeraldas in 2002 and 2013. No mutations in *Pfdhps* have ever been previously reported in Ecuador [25, 26].

Despite of the presence of mutations in *Pfdhfr* and *Pfdhps* in samples from Ecuador, the high prevalence of wild type genotypes suggests ongoing sensitivity to SP in the country. In 2002, 90% of samples collected in Esmeraldas presented at least one mutation in *Pfdhfr* (position 108 N). In addition, double (positions 108**N**, 164**L**) and triple (51**I**, 108**N**, 164**L**) *Pfdhfr* mutations were reported [25]. The decrease in the frequency of parasites carrying *Pfdhfr* mutations in Esmeraldas province could be related to the change in treatment from artesunate + SP to ATM-LUMF, which reduced the parasites from drug pressure.

*Pfmdr1* codes for a transmembrane P-glycoprotein in the DV of the parasites involved in transport of substrates from the cytosol to the DV. This protein belongs to the adenosine triphosphate-binding cassette transporter family [12]. Two factors have been associated with alteration of function in PfMDR1: mutations present in *Pfmdr1* and copy number increase [32]. The mutations N86**Y** and Y184**F** are more common in Asia and Africa. In contrast, in South America the mutations S1034**C**, N1042**D** and D1246**Y** are found to be more common [13]. These mutations are associated with multidrug resistance [37].

In Ecuador, the mutations 184**F** and 1042**D** were found frequently and were present in the majority of samples from an outbreak that occurred in Esmeraldas in 2013 [26]. These double mutants were found in this study in samples from Esmeraldas, San Lorenzo, Carchi and Sucumbíos. Furthermore, this genotype was found in Orellana and previously reported for the Ecu 1110 parasite [27]. The *Pfmdr1* polymorphisms (86, 1034, 1042, 1246) have been associated with resistance to QN, MQ, DHA, and HF [12]. The 184**F** mutation has not been associated with any specific drug resistance and the mutation 1042**D** has been linked to MQ and QN resistance [32]. Ecuadorian parasites do not present clinical or in vitro resistance to MQ or QN, suggesting that these drugs can be considered as an alternative to current treatment in the future.

The increase in *Pfmdr1* copy number has been associated with *P. falciparum* resistance to MQ, QN and ART [14–16]. All Ecuadorian *P. falciparum* parasites in this study showed one copy of this gene, suggesting that these parasites are sensitive to MQ. Efficacy in vivo studies of artesunate and MQ combination showed that these drugs were an effective treatment in Ecuador in 2000 [38]. In South America, there are reports of modifications in copy number in samples from the Pacific region, Atlantic region and southeast of Colombia (2009–2012), where 32% of the isolates had *Pfmdr1* copy numbers increase to two to five copies [15]. Peru reported single copy number for *Pfmdr1* in 2009 in the Amazon region [17].

The mutation 1042**D** and the increase in copy number have been associated with MQ resistance [32]. MQ forms hydrogen bonds with the residue 1042 of PfMDR1 and the change of N (asparagine) to D (acid aspartic) in this position may result in the inhibition of MQ passage through the DV membrane [39]. In order to test this hypothesis, live cell imaging using Fluo 4 AM was performed.

Fluo 4 AM is a fluorochrome that has been used to determine PfMDR1 transport of substrates from the cytosol to the DV of the parasite [32]. The parasites that present N1042 (wild type genotype) show an increased Fluo-4 fluorescence in the DV, showing that the fluorochrome is readily transported into this compartment [30, 32, 33]. In contrast, the parasites with 1042**D** (mutated) PfMDR1 show no increase in Fluo-4 fluorescence in the DV (there is rather an increase in fluorescence in the cytosol of the parasites), suggesting that the fluorochrome is not transported into the DV [30, 32, 33].

The mutation N1042**D** was found in most Ecuadorian *P. falciparum* isolates tested in this study, including ESM-2013. The Fluo 4 AM assay was performed in the isolate ESM-2013 from Esmeraldas to confirm that this mutation inhibited the transport of this marker. The fluorescence intensity of Fluo-4 in the DV of Dd2 (N1042) was higher than in ESM-2013. The Ecuadorian *P. falciparum* presented the mutation 1042**D** and inhibited the transport of Fluo 4 into the DV and did not present in vitro resistance to MQ. This suggests that resistance to MQ can be related to the synergy between polymorphisms and increase in copy number of *Pfmdr1*. The results showed that PfMDR1 of ESM-2013 is not completely functional, since there was inhibition of Fluo 4 transport. These results are not directly related with the current treatment for *P. falciparum* in Ecuador but should be considered in case a treatment change is planned.

*Plasmodium falciparum* resistance to ART has been reported in Southeast Asian countries, particularly in the Grand Mekong area (China, Vietnam, Cambodia, Thailand, Myanmar). ART resistance has been associated with mutations in *k13* [18, 19, 21, 40]. All Ecuadorian samples presented the wild type CRYGI (positions 476, 493, 539, 543, 580) genotype for this gene. Similarly, recent studies in Brazilian, Peruvian and Colombian isolates have shown a lack of *k13* mutations and the ACT treatment appears to be effective in these South American regions. The mutation C580**Y** (associated with ART susceptibility in Southeast Asia) was found in 5% of *P. falciparum* isolates from Guyana, even though ART showed 100% efficacy [20]. Although no ATM resistance mutations were found in the studied samples, the spread of other mutations related to ART resistance cannot be ruled out.

Drug resistance phenotypes can also be characterized by in vitro assays. In this study, in vitro assays were used to associate drug susceptibility phenotypes to drug-resistant genotypes. In vitro studies are used to monitor the drug susceptibility of *P. falciparum* and help guide the drug policy in each country. These studies can give a better idea of how the interaction between the parasite and the drug occur and can help establish parasite sensitivity.

Ecuadorian parasites were cultured and exposed to common anti-malarial drugs to establish their drug susceptibility. The ESM-2013 parasite showed an IC_50_ of 93.7 nM, confirming a CQ-resistant phenotype in Ecuadorian *P. falciparum* isolates having the mutation K76**T**. The in vitro resistance to CQ in Ecuadorian parasites has been previously reported in Ecu1110. It presented an IC_50_ > 90.9 nM [27] in comparison with 3D7 that showed an IC_50_ < 10 nM. Other in vitro studies have shown resistance to CQ in Colombia, where 90% of parasites analysed presented IC_50_ > 100 nM [22]. CQ resistance has been present in Ecuador since the 1980s, suggesting that the resistance to CQ is fixed in Ecuadorian *P. falciparum* parasites. ESM-2013 showed in vitro sensitivity to QN, MQ, DHA, LUMF, and ATM; similarly, Colombian samples had in vitro sensitivity to DHA, LUMF and ATM [15].

The current treatment for *P. falciparum* in Ecuador is ATM-LUMF. The results of this study suggest that this treatment continues to be effective in the country, as well as in the rest of Latin America where there is no reported resistance to ACT treatment [5]. It is important to note that, even though Ecuadorian parasites have CQ-resistant genotype and phenotype and present mutations in *Pfdhfr* and *Pfmdr1*, they have the same resistance profile as Ecu 1110, an isolate collected in 1990 [26]. These results suggest that the mutations in drug resistance genes have been maintained for almost 30 years in spite of a lack of selective pressure. This could be explained by a fixation of drug resistant mutations and the presence of parasites as asymptomatic reservoirs [41].

## Conclusion

This study shows that Ecuadorian *P. falciparum* had chloroquine-resistant genotype and phenotype, but were sensitive to sulfadoxine, pyrimethamine, artemether, lumefantrine, quinine, mefloquine, and dihydroartemisinin, indicating that the status of anti-malarial resistance during 2013–2015 remained effective. Nevertheless, this study will need to be completed with samples from the Amazon region to clarify the situation of resistance in all endemic areas of Ecuador. This work shows the importance of combining molecular and in vitro studies as surveillance tools to aid malaria elimination in the country. Early detection of resistance to the current drug treatment used for *P. falciparum* infections is essential to hinder outbreaks. This study presents the status of anti-malarial resistance in Ecuador and informs malaria elimination campaigns in the country.

## Supplementary information

**Additional file 1: Table S1.** Primers and conditions used for amplification of drug resistance markers.

## Data Availability

The datasets used and/or analyzed during the current study are available from the corresponding author on reasonable request

## References

[1] World Health Organization (2016). World malaria report.

[2] MSP (2017). Gaceta Epidemiológica Semanal No. 52: Ministerio de Salud Pública del Ecuador.

[3] PAHO (2016). Report on the situation of malaria in the Americas, 2000–2015.

[4] WHO (2018). World malaria report.

[5] Recht J, Siqueira AM, Monteiro WM, Herrera SM, Herrera S, Lacerda MVG (2017). Malaria in Brazil, Colombia, Peru and Venezuela. Current challenges in malaria control and elimination. Malar J..

[6] Fidock DA, Nomura T, Talley AK, Cooper RA, Dzekunov SM, Ferdig MT (2000). Mutations in the *P. falciparum* digestive vacuole transmembrane protein PfCRT and evidence for their role in chloroquine resistance. Mol Cell..

[7] Pelleau S, Moss EL, Dhingra SK, Volney B, Casteras J, Gabryszewski SJ (2015). Adaptive evolution of malaria parasites in French Guiana: reversal of chloroquine resistance by acquisition of a mutation in pfcrt. Proc Natl Acad Sci USA.

[8] Cortese JF, Caraballo A, Contreras CE, Plowe CV (2002). Origin and dissemination of *Plasmodium falciparum* drug-resistance mutations in South America. J Infect Dis.

[9] Corredor V, Murillo C, Echeverry DF, Benavides J, Pearce RJ, Roper C (2010). Origin and dissemination across the Colombian Andes mountain range of sulfadoxine-pyrimethamine resistance in *Plasmodium falciparum*. Antimicrob Agents Chemother.

[10] Griffing SM, Mixson-Hayden T, Sridaran S, Alam MT, McCollum AM, Cabezas C (2011). South American *Plasmodium falciparum* after the malaria eradication era: clonal population expansion and survival of the fittest hybrids. PLoS ONE.

[11] Sridaran S, Rodriguez B, Mercedes Soto A, Macedo De Oliveira A, Udhayakumar V (2014). Molecular analysis of chloroquine and sulfadoxine-pyrimethamine resistance-associated alleles in *Plasmodium falciparum* isolates from Nicaragua. Am J Trop Med Hyg..

[12] Price RN, Uhlemann A-C, Brockman A, McGready R, Ashley E, Phaipun L (2004). Mefloquine resistance in *Plasmodium falciparum* and increased pfmdr1 gene copy number. Lancet.

[13] Veiga MI, Dhingra SK, Henrich PP, Straimer J, Gnädig N, Uhlemann A-C (2016). Globally prevalent PfMDR1 mutations modulate *Plasmodium falciparum* susceptibility to artemisinin-based combination therapies. Nat Commun..

[14] Ferreira ID, do Rosário VE, Cravo PV (2006). Real-time quantitative PCR with SYBR Green I detection for estimating copy numbers of nine drug resistance candidate genes in *Plasmodium falciparum*. Malar J..

[15] Aponte S, Patricia Guerra Á, Álvarez-Larrotta C, Bernal S, Restrepo C, González C (2017). Baseline in vivo, ex vivo and molecular responses of *Plasmodium falciparum* to artemether and lumefantrine in three endemic zones for malaria in Colombia. Trans R Soc Trop Med Hyg.

[16] Costa GL, Amaral LC, Fontes CJF, Carvalho LH, de Brito CFA, de Sousa TN (2017). Assessment of copy number variation in genes related to drug resistance in *Plasmodium vivax* and *Plasmodium falciparum* isolates from the Brazilian Amazon and a systematic review of the literature. Malar J..

[17] Bacon DJ, McCollum AM, Griffing SM, Salas C, Soberon V, Santolalla M (2009). Dynamics of malaria drug resistance patterns in the Amazon Basin region following changes in Peruvian national treatment policy for uncomplicated malaria. Antimicrob Agents Chemother.

[18] Straimer J, Gnädig NF, Witkowski B, Amaratunga C, Duru V, Ramadani AP (2015). K13-propeller mutations confer artemisinin resistance in *Plasmodium falciparum* clinical isolates. Science.

[19] Cheeseman IH, Miller BA, Nair S, Nkhoma S, Tan A, Tan JC (2012). A major genome region underlying artemisinin resistance in malaria. Science.

[20] Chenet SM, Schneider KA, Villegas L, Escalante AA (2012). Local population structure of *Plasmodium*: impact on malaria control and elimination. Malar J..

[21] Chenet SM, Okoth SA, Kelley J, Lucchi N, Huber CS, Vreden S (2017). Molecular profile of malaria drug resistance markers of *Plasmodium falciparum* in Suriname. Antimicrob Agents Chemother.

[22] Aponte SL, Díaz G, Pava Z, Echeverry DF, Ibarguen D, Rios M (2011). Sentinel network for monitoring in vitro susceptibility of *Plasmodium falciparum* to antimalarial drugs in Colombia: a proof of concept. Mem Inst Oswaldo Cruz.

[23] Segurado A, Di Santi S, Shiroma M (1997). In vivo and in vitro *Plasmodium falciparum* resistance to chloroquine, amodiaquine and quinine in the Brazilian Amazon. Rev Inst Med Trop São Paulo..

[24] White J, Mascarenhas A, Pereira L, Dash R, Walke JT, Gawas P (2016). In vitro adaptation of *Plasmodium falciparum* reveal variations in cultivability. Malar J..

[25] Arróspide N, Hijar-Guerra G, de Mora D, Diaz-Cortéz CE, Veloz-Perez R, Gutierrez S (2014). Alelos mutantes asociados a la resistencia a cloroquina y sulfadoxina-pirimetamina en *Plasmodium falciparum* de las fronteras Ecuador-Perú y Ecuador-Colombia. Rev Peru Med Exp Salud Pública..

[26] Sáenz FE, Morton LC, Okoth SA, Valenzuela G, Vera-Arias CA, Vélez-Álvarez E (2015). Clonal population expansion in an outbreak of *Plasmodium falciparum* on the northwest coast of Ecuador. Malar J..

[27] Sá JM, Twu O, Hayton K, Reyes S, Fay MP, Ringwald P (2009). Geographic patterns of *Plasmodium falciparum* drug resistance distinguished by differential responses to amodiaquine and chloroquine. Proc Natl Acad Sci USA.

[28] Moll K, Kaneko A, Scherf A, Wahlgren M (2013). Methods in malaria research.

[29] Snounou G (1996). Detection and identification of the four malaria parasite species infecting humans by PCR amplification. Methods Mol Biol.

[30] Friedrich O, Reiling SJ, Wunderlich J, Rohrbach P (2014). Assessment of *Plasmodium falciparum* PfMDR1 transport rates using Fluo-4. J Cell Mol Med.

[31] Schuster FL (2002). Cultivation of *Plasmodium* spp. Clin Microbiol Rev.

[32] Reiling SJ, Rohrbach P (2015). Monitoring PfMDR1 transport in *Plasmodium falciparum*. Malar J..

[33] Rohrbach P, Sanchez CP, Hayton K, Friedrich O, Patel J, Sidhu ABS (2006). Genetic linkage of pfmdr1 with food vacuolar solute import in *Plasmodium falciparum*. EMBO J.

[34] MSP. Situación de la malaria en el Ecuador. Gaceta Ministerio de Salud Pública del Ecuador. Quito, 2013.

[35] WHO (2018). Country epidemiological profile: Ecuador.

[36] Vera-Arias CA, Castro LE, Gómez-Obando J, Sáenz FE (2019). Diverse origin of *Plasmodium falciparum* in northwest Ecuador. Malar J..

[37] Li J, Chen J, Xie D, Monte-Nguba S, Eyi JUM, Matesa RA (2014). High prevalence of pfmdr1 N86Y and Y184F mutations in *Plasmodium falciparum* isolates from Bioko island, Equatorial Guinea. Pathog Glob Health..

[38] Gomez LEA, Jurado MH, Cambon N (2003). Randomised efficacy and safety study of two 3-day artesunate rectal capsule/mefloquine regimens versus artesunate alone for uncomplicated malaria in Ecuadorian children. Acta Trop.

[39] Patel SK, George L-B, Prasanth Kumar S, Highland HN, Jasrai YT, Pandya HA (2013). A Computational approach towards the understanding of *Plasmodium falciparum* multidrug resistance protein 1. ISRN Bioinforma..

[40] Sidhu ABS, Uhlemann A-C, Valderramos SG, Valderramos J-C, Krishna S, Fidock DA (2006). Decreasing pfmdr1 copy number in *Plasmodium falciparum* malaria heightens susceptibility to mefloquine, lumefantrine, halofantrine, quinine, and artemisinin. J Infect Dis.

[41] Sáenz FE, Arévalo-Cortés A, Valenzuela G, Vallejo AF, Castellanos A, Poveda-Loayza AC (2017). Malaria epidemiology in low-endemicity areas of the northern coast of Ecuador: high prevalence of asymptomatic infections. Malar J..

[42] Ladeia-Andrade S, de Melo GNP, de Souza-Lima RD, Salla LC, Bastos MS, Rodrigues PT (2016). No clinical or molecular evidence of *Plasmodium falciparum* resistance to artesunate–mefloquine in Northwestern Brazil. Am J Trop Med Hyg..

