# Supplementary figures and images for: Genotypes and phenotypes of resistance in Ecuadorian Plasmodium falciparum
Source: Malar J. 2019 Dec 10;18:415. doi: 10.1186/s12936-019-3044-z (PMC6905098; doi:10.1186/s12936-019-3044-z)

**
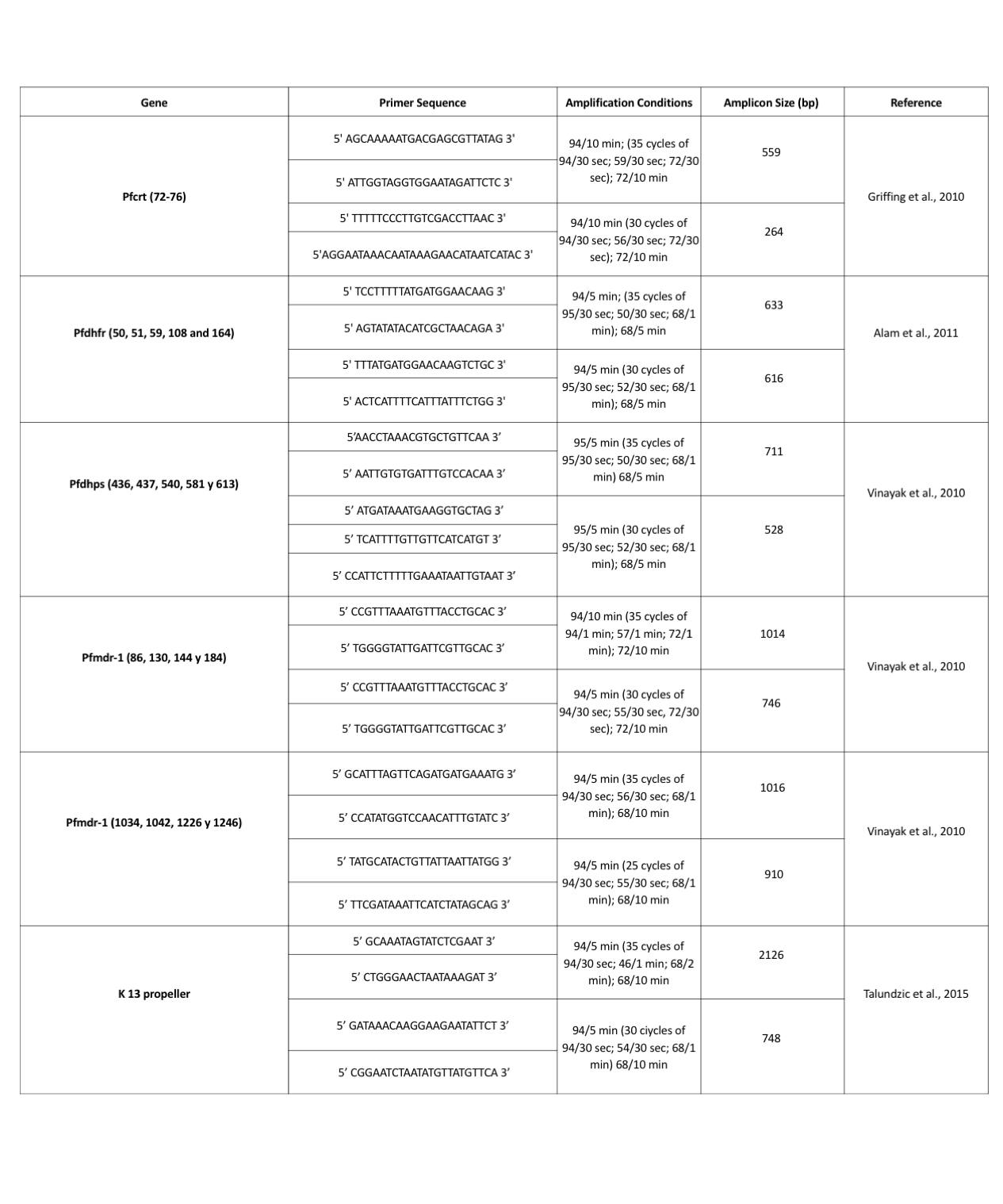
Table S1 Primers and conditions used for amplification of drug resistance markers**

Supplement: Supplementary file 1 — Additional file 1: Table S1. Primers and conditions used for amplification of drug resistance markers. [file 12936_2019_3044_MOESM1_ESM.docx]
